# Construction of PCN-222 and Atomically Thin 2D CNs Van Der Waals Heterojunction for Enhanced Visible Light Photocatalytic Hydrogen Production

**DOI:** 10.3390/nano13081318

**Published:** 2023-04-09

**Authors:** Liting Wu, Xuke Mi, Shaopeng Wang, Can Huang, Yu Zhang, Yong-Mei Wang, Yong Wang

**Affiliations:** 1 School of Advanced Materials and Nanotechnology, Academy of Advanced Interdisciplinary Research, Xidian University, Xi’an 710126, China; 2 Department of Physics, Shaanxi University of Science and Technology, Xi’an 710021, China; 3 The State Key Discipline Laboratory of Wide Band Gap Semiconductor Technology, Xidian University, Xi’an 710126, China

**Keywords:** PCN-222, two-dimensional carbon nitride, van der Waals heterostructures, hydrogen production

## Abstract

Atomically thin two-dimensional (2D) CN sheets have attracted extensive attention in the field of photocatalysis because of their shorter diffusion path of photogenerated carriers and abundant surface reaction sites than bulk CN. However, 2D CNs still exhibit poor visible-light photocatalytic activity because of a strong quantum size effect. Here, PCN-222/CNs vdWHs were successfully constructed using the electrostatic self-assembly method. The results showed that PCN-222/CNs vdWHs with 1 wt.% PCN-222 enhanced the absorption range of CNs from 420 to 438 nm, which improved the absorption capacity of visible light. Additionally, the hydrogen production rate of 1 wt.% PCN-222/CNs is four times that of the pristine 2D CNs. This study provides a simple and effective strategy for 2D CN-based photocatalysts to promote visible light absorption.

## 1. Introduction

Direct solar energy conversion into hydrogen fuel using semiconductor photocatalysts has been seen as one of the promising ways to address problems, such as natural environmental pollution and global energy shortage [1,2,3,4]. Carbon nitride (CN), as an ideal metal-free semiconductor photocatalyst, has attracted extensive attention since it was reported for visible-light photocatalytic hydrogen production in 2009 [5,6,7]. Its advantages include an excellent electronic band structure for the production of hydrogen, a narrow bandgap, excellent physicochemical stability, facile synthesis and easy functionalization [8,9,10,11,12,13]. In particular, atomically thin two-dimensional (2D) CN sheets (CNs) have a shorter diffusion path of photogenerated carriers than bulk CN, abundant surface reaction sites and more effective separation of internal photogenerated carriers [14,15,16]. Therefore, among various semiconductor photocatalysts, atomically thin 2D CNs have become a rising star in the field of photocatalysis. However, 2D CNs still exhibit poor visible-light photocatalytic activity because of a strong quantum size effect that makes visible light more difficult to capture [17,18]. Therefore, the primary challenge in developing 2D CN-based photocatalysts is to increase their visible light absorption.

Fortunately, creating van der Waals heterojunctions (vdWHs) to achieve enhanced visible light absorption of 2D CNs is a simple and effective approach [19,20]. The effect of interlayer distance will be affected by the construction of vdWHs, thereby influencing the band gap to promote charge separation and transfer, which is beneficial to catalysis [21,22]. In order to construct a high-efficiency heterostructure, it is crucial to have a well-matched energy band. In recent years, metal-organic frameworks (MOFs) have attracted considerable attention in the field of photocatalysis due to their abundance of reaction sites, large surface area and good thermal stability [23,24,25,26]. More significantly, among the vast variety of MOFs, PCN-222 has a narrow bandgap of about 1.69–1.74 eV, that can be well matched with the wide bandgap 2D CNs (3.06 eV) [27,28]. Furthermore, as a result of its coordination relationship between Zr_6_(O)_4_(OH)_4_ nodes and tetrakis(4-carboxyphenyl)-porphyrin (H_2_TCPP) links, PCN-222 exhibits improved adsorption and reaction capabilities in visible light [29]. Therefore, inspired by these studies, we build PCN-222/CNs vdWHs using the electrostatic self-assembly method to enhance the visible light absorption of 2D CNs. The experimental results showed that the introduction of 1 wt.% PCN-222 enhanced the absorption range of CNs from 420 nm to 438 nm, which improved the absorption capacity of visible light. In addition, the photocatalytic hydrogen production activity of PCN-222/CNs vdWHs is four times higher than that of pure CNs with excellent stability.

## 2. Materials and Methods

### 2.1. Materials

The urea (99.999%), ZrCl_4_, tetrakis(4-carboxyphenyl)-porphyrin (TCPP), benzoic acid and ethanol were all purchased from Aladdin (Shanghai, China). N, N-dimethylformamide (DMF, AR) was obtained from Fuyu Chemical (Tianjin, China). Deionized water was acquired from the SMART water purification system. All of the reagents were AR or higher grade.

### 2.2. Synthesis of Atomically Thin 2D CNs

In order to synthesize bulk CN, a crucible with a lid was wrapped with aluminum foil. The crucible contained approximately 10 g of urea (Aladdin, 99.999%). The urea was then heated to 550 °C for 3 h in an air atmosphere in a muffle furnace. Then, 150 mg of bulk CN were measured and placed in an uncovered ceramic container (12 cm × 6 cm × 1 cm). Then, the bulk CN was heated at 530 °C for 2 h. Ultimately, 2D CNs with atomically thin were manufactured.

### 2.3. Synthesis of PCN-222

PCN-222 was prepared using the solvothermal method. Thirty mg of ZrCl_4_ and 10 mg of TCPP were dissolved in 12 mL N,N-dimethylformamide (DMF) with ultrasonic assistance. Then, 400 mg of benzoic acid was added to the solution. Once the solution was completely dissolved by ultrasound, it was heated in an oven to 120 °C for 24 h. The product was washed with DMF, ethanol and deionized water, respectively. Finally, PCN-222 was obtained by freeze-drying.

### 2.4. Synthesis of PCN-222/CNs Van Der Waals Heterojunction

The electrostatic adsorption self-assembly technique was used to build a PCN-222/CNs van der Waals heterojunction. Firstly, 150 mg of 2D CNs were added to 150 mL of hydrochloric acid aqueous solution and ultrasonically oscillated for 1 h, and the pH of the solution was 4. Then, 1.5 mg, 3 mg and 4.5 mg of PCN-222 were added to the solution, respectively. Following stirring for 24 h, PCN-222/CNs vdWHs was achieved by creating electrostatic adsorption between PCN-222 and CNs, which are named X wt.% PCN-222/CNs (X = 1, 3, 5).

### 2.5. Characterization

X-ray powder diffraction (XRD) was recorded on a Bruker D8 ADVANCE (Karlsruhe, Germany,). Fourier transform infrared (FT-IR) spectra was obtained using the Thermo Scientific Nicolet iS50 (Waltham, MA, USA). Transmission electron microscope (TEM) and energy dispersive spectroscopy (EDS) were recorded on a JEM-2100F microscope (Tokyo, Japan). X-ray photoelectron spectroscopy (XPS) measurements were collected using a Thermo Scientific K-Alpha+ (Waltham, MA, USA). In addition, the binding energies were calibrated by the C 1s peak at 284.6 eV. UV–visible absorption spectroscopy (UV–vis) absorption data were recorded by a Perkin Elmer Lambda UV–vis spectrophotometer (Waltham, MA, USA). Photoluminescence emission spectra (PL) were performed using Edinburgh FLS980 (Edinburgh, Scotland, UK).

### 2.6. Photocatalytic Activity Measurement

The 100 mL aqueous solution with 10% triethanolamine (TEOA) was mixed with 25 mg of PCN-222/CNs vdWHs, where the samples were sonicated for 30 min in the reaction system. Then, the hydrogen evolution of PCN-222/CNs vdWHs photocatalyst using 3% Pt as cocatalyst occurred. As the illumination source, a Xenon 300 W lamp (MC-PF300C, λ > 420 nm) was employed. The distance between the light source and the reactor was 2 cm. The temperature of the injector and the column was 129 °C and 100 °C, respectively. The reaction system was monitored with a GC9720PLUS gas chromatography thermal conductivity detector. The high-purity argon was used as carrier gas. Then, the samples were illuminated for 4 h, with one data point captured each hour.

## 3. Results and Discussions

**Figure 1:**
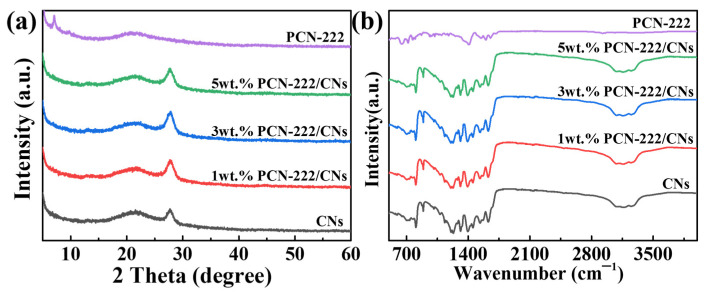
(**a**) XRD patterns and (**b**) FT-IR patterns of PCN-222, CNs and PCN-222/CNs.

The crystal structures of PCN-222, 2D CNs and PCN-222/CNs were investigated using XRD, and the findings are depicted in Figure 1a. In the XRD pattern of the CNs, there is an obvious peak at 27.8°, which corresponds to the (002) crystal plane of the CNs [30]. The XRD pattern of PCN-222 shows the major diffraction peaks at 7.1° and 9.8°, that are related to the characteristic peaks of PCN-222 [31]. These results indicated that CNs and PCN-222 were successfully prepared. In the XRD pattern of PCN-222/CNs, the characteristic peaks of CNs were clearly observed, indicating that the introduction of PCN-222 did not destroy the structure of the CNs. However, the characteristic peaks of PCN-222 were hardly observed, which may be caused by a low percentage of PCN-222 and a large area of dispersion. Figure 1b is the FT-IR spectra of PCN-222, CNs and PCN-222/CNs. A characteristic peak at 806 cm^−1^ can be found in the CNs, that is associated with the stretching vibration of tri-s-triazine rings [32]. The peaks between 900 and 1800 cm^−1^ were attributed to the typical C-N heterocycles [33]. In addition, the characteristic peaks in the range of 3000–3300 cm^−1^ originated from the N-H stretching vibrations [32,33]. The FT-IR spectra of the CNs further indicates that CNs were successfully prepared, matching the results of XRD. The characteristic vibration peaks at 719.1 cm^−1^, 767 cm^−1^, 806.8 cm^−1^, 868 cm^−1^, 1010.6 cm^−1^, 1177.5 cm^−1^, 1344 cm^−1^, 1407.5 cm^−1^, 1556.1 cm^−1^, 1601.8 cm^−1^ and 1714.5 cm^−1^ have a minor shift compared with the other literature on PCN-222, which may be due to the presence of solvent molecule residues [34,35,36]. In the FT-IR spectra of PCN-222/CNs, it is noted that the characteristic peaks belonging to the CNs could be observed, but no obvious characteristic peaks of PCN-222 could be detected. This phenomenon occurred because the load of PCN-222 in the heterojunction was too low and the peak intensity of PCN-222 was too weak. However, compared with the CNs, the characteristic absorption peak of PCN-222/CNs have a minor shift of about 5 cm^−1^ towards the direction of low wavenumbers, that indicates the presence of interactions at the interface of PCN-222 and CNs, and combining by van der Waals [21].

**Figure 2:**
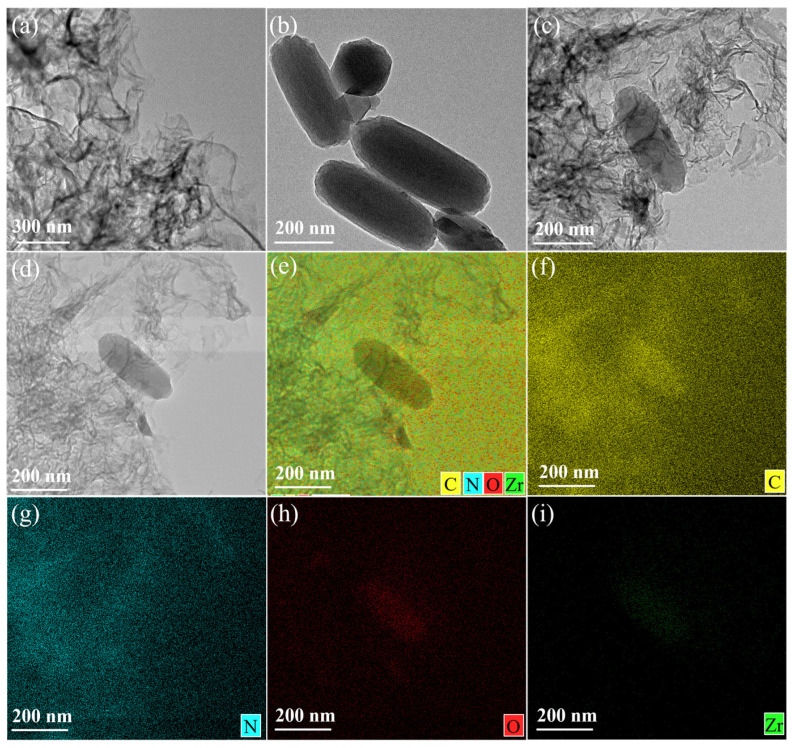
TEM images of (**a**) 2D CNs, (**b**) PCN-222 and (**c**) PCN-222/CNs. (**d**–**i**) TEM-EDS elemental mapping of PCN-222/CNs.

The morphological structure of PCN-222, 2D CNs and PCN-222/CNs were characterized by TEM (Figure 2). According to Figure 2a, it is clear that 2D CNs display almost transparent flake morphologies, demonstrating their atomically thin thickness. PCN-222 is a relatively uniform rod-like structure with a length of about 300–500 nm (Figure 2b). Upon constructing the van der Waals heterojunction, PCN-222 was wrapped in 2D CNs (Figure 2c), indicating an effective interfacial contact between 2D CNs and PCN-222 to enhance the photocatalytic performance [36]. Furthermore, the presence of carbon, nitrogen, oxygen and zirconium in PCN-222/CNs from the EDS images clearly demonstrates the successful construction of the van der Waals heterojunction (Figure 2d–i). The results of TEM further prove the successful construction of van der Waals, which is consistent with the above XRD and FT-IR results.

**Figure 3:**
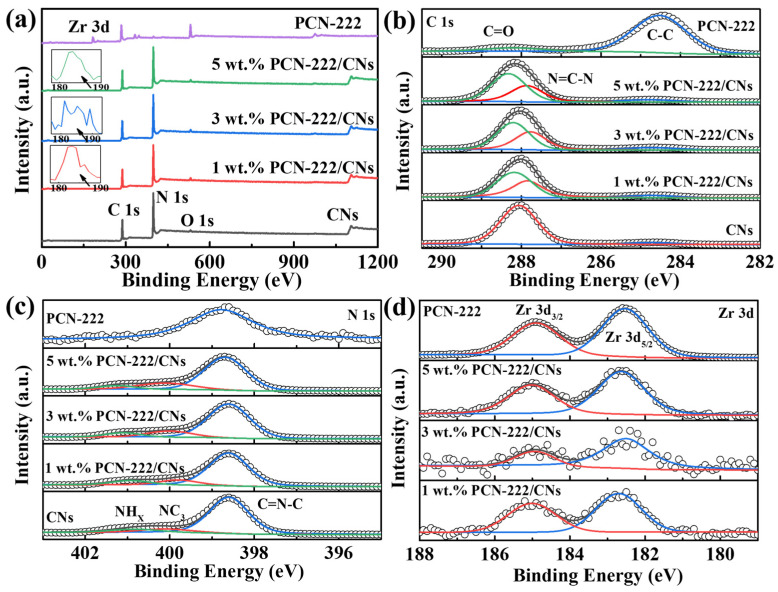
XPS spectra of PCN-222, CNs and PCN-222/CNs. (**a**) Survey, (**b**) C 1s, (**c**) N 1s, (**d**) Zr 3d.

The chemical states and chemical composition of PCN-222, CNs and PCN-222/CNs were investigated using the XPS technique, as depicted in Figure 3. It can be observed that the 2D CNs mainly contain C and N elements, and a small amount of O elements come from the surface absorbed H_2_O or O_2_ (Figure 3a) [37]. There are four elements of C, N, O and Zr in the PCN-222. For the PCN-222/CNs, although the very weak O 1s and Zr 3d signals, the existence of C, N, O and Zr elements is still confirmed. This is due to the lower content of PCN-222, further demonstrating the successful construction of the PCN-222/CNs vdWHs. As presented in Figure 3b, C 1s contains the two peaks at 284.6 eV and 287.9 eV, that corresponds to the C-C bond and the N=C-N bond, respectively [28]. Two main peaks at 284.6 eV and 288.3 eV can be found in the sample of PCN-222, that are related to C-C and C=O, respectively [36]. Notably, the N=C-N and C=O in the samples of PCN-222/CNs exhibited a negative shift, proving that CNs and PCN-222 have a strong interaction. The peak at 298.7 eV corresponds to C=N-C bond for PCN-222, as shown in Figure 3c. N 1s contains three peaks at 398.6 eV, 400.0 eV and 400.8 eV, which are attributed to C=N-C, NC_3_ and NH_X_ for CNs, respectively [28]. Interestingly, these groups were slightly shifted for PCN-222/CNs, further demonstrating the strong interaction at the heterojunction interface. In the Zr 3d pattern (Figure 3d), the peaks at 182.5 eV and 184.9 eV can be observed in PCN-222, that corresponds to Zr 3d_5/2_ and Zr 3d_3/2_, respectively [36,38]. Compared to the PCN-222, the bonds in PCN-222/CNs were slightly shifted, which proves the strong interaction between CNs and PCN-222, and the successful construction of the van der Waals heterojunctions.

**Figure 4:**
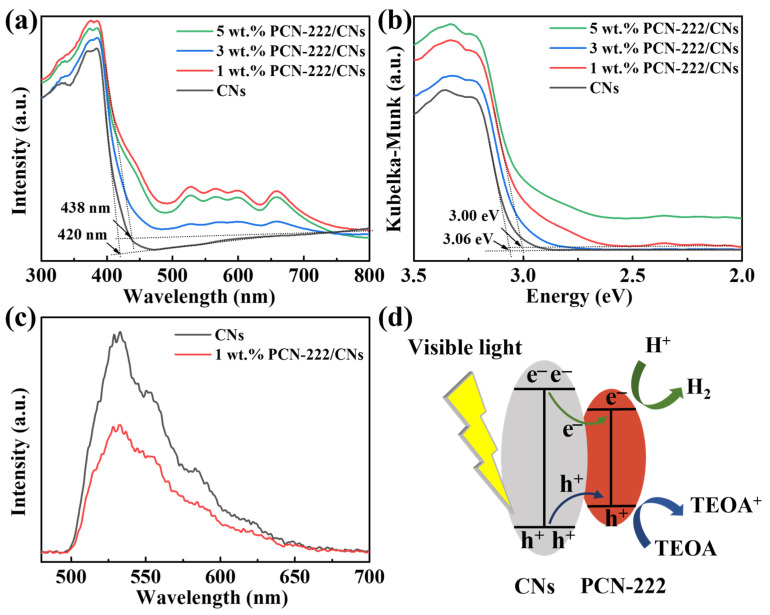
(**a**) UV–Vis spectra and (**b**) the corresponding (αhν)^1/2^ curves of CNs and PCN-222/CNs. (**c**) PL spectra of CNs and 1 wt.% PCN-222/CNs with a 325 nm excitation wavelength. (**d**) Proposed photocatalytic hydrogen evolution reaction mechanism of PCN-222/CNs vdWHs.

The optical absorption property of CNs and PCN-222/CNs were analyzed by UV–Vis, and the results can be observed in Figure 4. It can be seen that the sample of CNs in Figure 4a displays an absorption edge at 420 nm, which is the result of electrons shifting from N 2p to C 2p orbitals [36]. Then, it is calculated that the band gap is 3.06 eV, which is in agreement with previous studies (Figure 4b) [15,30]. When PCN-222 was introduced to construct the vdWHs, it was observed that the absorption edge of the sample was improved. When the content of PCN-222 was 1%, the maximum absorption edge of the sample increased to 438 nm. However, the absorption edge decreased when adding 3% or 5% PCN-222. This is because if excessive amounts of PCN-222 is introduced, PCN-222 may aggregate and cover the surface of CNs, and then compete with CNs to absorb visible light [39]. In addition, the corresponding band gap of 1 wt.% PCN-222/CNs is calculated to 3.00 eV. According to the results of UV–Vis, we can conclude that the introduction of PCN-222 can narrow the band gap and enhance the absorption of visible light photons, which will benefit the photocatalytic reaction. Furthermore, to further explore the mechanism of the enhanced photocatalytic activity of the PCN-222/CNs vdWHs, PL tests were performed as depicted in Figure 4c. It can be observed that the emission peaks of PCN-222/CNs vdWHs and CNs have the same position, but the intensity is obviously different. Compared with the CNs, the strength of PCN-222/CNs vdWHs shows lower intensity, indicating that the addition of PCN-222 can improve the separation efficiency of electron-hole pairs [33,40]. The photocatalytic hydrogen evolution mechanism of the PCN-222/CNs vdWHs is proposed, as represented in Figure 4d. Under the irradiation of visible light, photogenerated electron-hole pairs can be generated both in CNs and PCN-222. Then, photogenerated electrons can be quickly transferred to the conduction band of PCN-222 through CNs and react with H_2_O to generate H_2_, while photoinduced holes are transferred and accumulated in the valence band of PCN-222 to oxidize TEOA to TEOA^+^ [39,40]. Therefore, the construction of PCN-222/CNs vdWHs can enhance the photocatalytic hydrogen evolution ability through the effective separation of charges.

**Figure 5:**
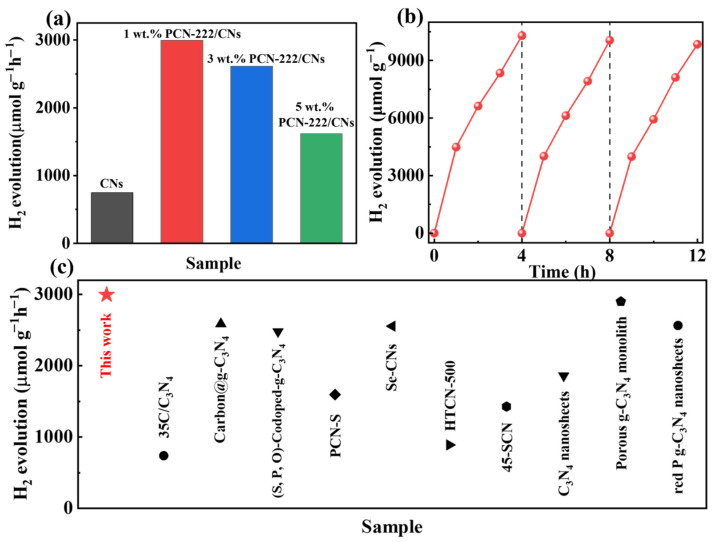
(**a**) Photocatalytic hydrogen production activity of CNs and PCN-222/CNs. (**b**) Cycling tests of 1 wt.% PCN-222/CNs. (**c**) Hydrogen production rate of some reported catalysts [41,42,43,44,45,46,47,48,49,50].

To study the performance of photocatalysis, we conducted a hydrogen production test on the samples in visible light. As illustrated in Figure 5a, 2D CNs showed a lower hydrogen production rate of 749.74 μmol g^−1^ h^−1^, that was attributed to a strong quantum size effect in 2D CNs that makes visible light more difficult to capture. With the introduction of PCN-222, a van der Waals heterojunction was formed with the CNs, and the hydrogen production rate was significantly improved. The sample with the highest hydrogen production rate was 1 wt.% PCN-222/CNs (2996.16 µmol g^−1^ h^−1^), which is four times that of the pristine 2D CNs. Notably, the photocatalytic efficiency decreased significantly when PCN-222 was in excess. There are two main reasons: (i) excessive PCN-222 may accelerate the recombination of electron-hole pairs; (ii) excessive PCN-222 may lead to agglomeration, covering some active sites and increasing the length of the electron transfer of CNs [39,41]. Furthermore, the cyclic stability of 1 wt.% PCN-222/CNs is investigated as depicted in Figure 5b. The results demonstrate that the hydrogen generation of 1 wt.% PCN-222/CNs still shows excellent performance after three cycles (every 4 h in one cycle). Additionally, compared with the photocatalysts in the previous literature, the PCN-222/CNs exhibited an outstanding ability for the H_2_ generation rate [41,42,43,44,45,46,47,48,49,50]. Therefore, the construction of the PCN-222/CNs vdWHs facilitates the visible light absorption and enhances the photocatalytic hydrogen evolution reaction.

## 4. Conclusions

In summary, PCN-222/CNs vdWHs were successfully constructed using the electrostatic self-assembly method. The experimental results showed that the introduction of 1 wt.% PCN-222 enhanced the absorption range of CNs from 420 nm to 438 nm, which improved the absorption capacity of visible light. PCN-222/CNs vdWHs with 1 wt.% PCN-222 showed an outstanding hydrogen production rate of 2996.16 µmol g^−1^ h^−1^, that is four times that of the pristine 2D CNs. The results show that the introduction of the PCN-222 facilitates the visible light absorption and enhances the photocatalytic hydrogen evolution reaction of 2D CNs. This study provides a simple and effective idea for future catalysts based on 2D CNs to promote visible light absorption.

## Data Availability

All of the relevant data are included in this published article.
